# Effects of Vitamin E on Bone Biomechanical and Histomorphometric Parameters in Ovariectomized Rats

**DOI:** 10.1155/2013/825985

**Published:** 2013-09-05

**Authors:** Rafaela G. Feresin, Sarah A. Johnson, Marcus L. Elam, Jeong-Su Kim, Dania A. Khalil, Edralin A. Lucas, Brenda J. Smith, Mark E. Payton, Mohammed P. Akhter, Bahram H. Arjmandi

**Affiliations:** ^1^Nutrition, Food & Exercise Sciences, The Florida State University, Tallahassee, FL 32306, USA; ^2^Center for Advancing Exercise and Nutrition Research on Aging, Department of Nutrition, Food and Exercise Sciences, The Florida State University, 436 Sandels Building, Tallahassee, FL 32306, USA; ^3^Departments of Nutritional Sciences, Oklahoma State University, Stillwater, OK 74078, USA; ^4^Departments of Statistics, Oklahoma State University, Stillwater, OK 74078, USA; ^5^Osteoporosis Research Center, Creighton University, Omaha, NE 68131, USA

## Abstract

The present study examined the dose-dependent effect of vitamin E in reversing bone loss in ovariectomized (Ovx) rats. Sprague-Dawley rats were either Sham-operated (Sham) or Ovx and fed control diet for 120 days to lose bone. Subsequently, rats were divided into 5 groups (*n* = 12/group): Sham, Ovx-control, low dose (Ovx + 300 mg/kg diet; LD), medium dose (Ovx + 525 mg/kg diet; MD), and high dose (Ovx + 750 mg/kg diet; HD) of vitamin E and sacrificed after 100 days. Animals receiving MD and HD of vitamin E had increased serum alkaline phosphatase compared to the Ovx-control group. Bone histomorphometry analysis indicated a decrease in bone resorption as well as increased bone formation and mineralization in the Ovx groups supplemented with MD and HD of vitamin E. Microcomputed tomography findings indicated no effects of vitamin E on trabecular bone of fifth lumbar vertebrae. Animals receiving HD of vitamin E had enhanced fourth lumbar vertebra quality as evidenced by improved ultimate and yield load and stress when compared to Ovx-control group. These findings demonstrate that vitamin E improves bone quality, attenuates bone resorption, and enhances the rate of bone formation while being unable to restore bone density and trabecular bone structure.

## 1. Introduction

Ovarian hormone deficiency is a major risk factor for osteoporosis in women [1, 2]. Although there are several FDA-approved medications to either prevent or reverse osteoporosis, women continue to look for safer and more feasible preventative and therapeutic alternatives [3]. In postmenopausal women, the rate of bone turnover increases with the rate of bone resorption exceeding that of bone formation which results in net bone loss [4]. These events, in part, have been linked to the modulation of immune cell mediators (cytokines and prostaglandins) and oxygen-derived free radical (ODFR) formation either in the bone microenvironment or in the cells that serve as osteoclastic precursors such as those of the monocyte-macrophage lineage [5].

A plethora of local cytokines and lipid mediators such as interleukin (IL)-1, IL-6, tumor necrosis factor-alpha (TNF-*α*), lymphotoxin, leukotrienes, and prostaglandins of the E series (PGE) are involved in regulating bone metabolism [6–8]. Ovarian hormone deficiency is associated with increased activation of certain immune cells leading to an increase in inflammatory mediators, such as IL-1 [9, 10], IL-6 [9, 11], and in particular PGE_2_ [12–17], which is in part responsible for suppressing osteoblastic activity stimulating osteoclastic differentiation, and activity resulting in an increase in bone resorption [18].

Oxygen-derived free radicals are also formed by a number of activated immune cells, including monocytes, macrophages, and neutrophils [19], and have been reported to be increased in chronic inflammatory diseases [20], aging [21], and osteoporosis [22, 23]. *In vivo* and *in vitro* findings [22–24] indicate that excessive free radicals generated in the bone environment increase osteoclastogenesis and bone resorption resulting in reduced bone mineral density (BMD) and osteoporosis [25]. A role of oxidative stress in estrogen-deficiency-induced bone loss has also been demonstrated as ovariectomy significantly reduces two of the primary thiol antioxidants, glutathione and thioredoxin, in osteoclasts [26, 27].

Vitamin E, a fat-soluble antioxidant [26, 28, 29], has been of particular interest due to its ability to suppress the production of certain proinflammatory mediators such as PGE_2_, TNF-*α*, IL-1, and IL-6 [30–34] that have been linked to increased bone loss [6–8, 35], and protect against oxidative damage caused by ODFRs [29, 36]. The relationship between vitamin E and bone has been studied to some extent. Xu et al. [37] reported that vitamin E maintains normal bone growth and bone modeling in young animals and protects cartilage against cellular lipid peroxidation. They also found that supplemental vitamin E increased bone mass by lowering the concentrations of free radicals that stimulate bone resorption and suppress bone formation. Vitamin E also reduced the ODFR stimulation of osteoclastic bone resorption [22]. Furthermore, the administration of *α*-tocopherol was shown to suppress the production of ODFRs in the early stages of fracture healing in rabbits [38], increase the activity of antioxidant enzymes in Ovx rats [39], and enhance bone fracture healing in its early [40] and late phases [41] in Ovx rats and in male rats [42]. In addition, the results of a population study by Melhus et al. [43] indicated the important role of adequate intake of dietary vitamin E in reducing the risk of hip fracture in current smokers. Another cross-sectional study suggests that vitamin E may uncouple bone turnover in postmenopausal women, causing an increase in the rate of bone formation while having no effects on the rate of bone resorption [44]. Collectively, these observations suggest that vitamin E positively influences skeletal health.

Previously, we demonstrated that supplemental levels of vitamin E improve bone quality as well as augment bone matrix protein without altering bone density in old male mice [45] and orchidectomized (Orx) male rats [46], both of which are well-accepted models of male, age-related osteoporosis. Furthermore, we have observed that supplemental doses of vitamin E have protective effects on various bone parameters in hindlimb unloaded male rat model of osteoporosis but not in ambulatory control rats [47]. Additionally, Norazlina et al. [48] reported that vitamin E is able to maintain BMD in Ovx rats. These findings imply that animals under physical stress and hormonal deficiency may benefit from additional vitamin E in the diet. Thus, the purpose of this study was to investigate whether vitamin E dose-dependently reverses bone loss in an Ovx rat model of osteoporosis. 

## 2. Materials and Methods

### 2.1. Animals and Diets

Seventy-five 12-month-old female Sprague-Dawley rats were housed in an environmentally controlled animal laboratory upon arrival. Guidelines for the ethical care and treatment of animals from the Animal Care and Use Committee were strictly followed. After 5 days of acclimation, rats were either sham-operated (Sham; 1 group; *n* = 12) or ovariectomized (Ovx; 4 groups). All rats were maintained on a semi-purified casein-based diet (AIN 93M) containing basal levels of vitamin E (75 mg vitamin E/kg diet) for a period of 120 days to induce osteopenia [49]. Ovariectomized rats were then randomly divided into 4 groups (*n* = 12/group): Ovx control, and Ovx + 300 mg vitamin E/kg diet (low dose, LD), Ovx + 525 mg vitamin E/kg diet (medium dose, MD), Ovx + 750 mg vitamin E/kg diet (high dose, HD). The animals were pair-fed to the average food intake of Sham group and had free access to distilled deionized water. Rats were fed their respective dietary treatments for 100 days and then sacrificed at the end of the treatment period. 

### 2.2. Necropsy and Tissue Processing

One-hundred days after treatment rats were anesthetized with a mixture of ketamine/xylazine (60 and 3 mg/kg body weight, resp.), and whole body BMD as well as bone mineral content (BMC) were measured using dual energy X-ray absorptiometry (DXA; QDR-4500A Elite; Hologic, Waltham, MA, USA). Rats were then bled via the abdominal aorta. Blood samples were collected, and serum was separated by centrifugation at 1500 ×g for 20 min at 4°C. Aliquots of serum were frozen and kept at −20°C for later analyses. The tibiae and the lumbar vertebrae were removed for various analyses. The tibiae were stored in 70% ethanol for histomorphometry while fourth and fifth lumbar vertebrae (L4 and L5, resp.) were frozen at −20°C for biomechanical properties and microstructural analysis, respectively. The uterus of each rat was collected, blotted, and weighed to confirm the success of ovariectomy.

### 2.3. Serum Vitamin E Analysis

Serum *α*-tocopherol was determined using the method of Bieri et al. [50]. Briefly, samples and standards were extracted with hexane, evaporated under nitrogen, and reconstituted with diethylether/methanol (1 : 3, v/v). Alpha-tocopherol acetate solution (5 mg/mL) (Sigma Chemical Co., St. Louis, MO, USA) was added to 200 *μ*L of serum or *α*-tocopherol acetate (Sigma Chemical Co., St. Louis, MO, USA) which was used as an internal standard. Vitamin E was measured in duplicate via HPLC (Waters Corporation, Milford, MA, USA) using a Nova-Pak C18 column (3.9 × 150 mm, 4 *μ*m particle size; Waters Corporation), methanol : water (95 : 5, v/v) as the mobile phase, and absorbance was monitored at 290 nm. The intra- and interassay coefficients of variation were 4.3% and 4.6%, respectively.

### 2.4. Serum Alkaline Phosphatase (ALP) Activity

Serum ALP activity was determined colorimetrically using a kit from Sigma Diagnostics (St. Louis, MO, USA). This test was performed on a Cobas-Fara II Clinical Analyzer (Montclair, NJ, USA). ALP activity was measured from the time-dependent increase in absorbance at 410 nm due to formation of nitrophenol. The intra- and interassay coefficients of variation of this procedure were 1.9% and 2.8%, respectively.

### 2.5. Bone Histomorphometry

Quantitative histomorphometry of the right tibia was performed to examine the dynamics of bone changes and bone cell activities in response to the ovariectomy and treatments. Briefly, calcein (8 mg/kg; Sigma, St. Louis, MO, USA) was injected at 8 days and 1 day, before necropsy, in order to prelabel the skeleton of the rats. The tibiae were cut at 1 mm distal to the tibio-ibula junction (TFJ) and 19 mm proximal to the TFJ to obtain a proximal portion for cancellous bone histomorphometry and fixed in 70% ethanol. The proximal tibiae (5 *μ*m thick) were prestained in Villanueva stain, dehydrated in graded ethanol and acetone, and individually embedded in methyl methacrylate. Goldner-stained specimen was used for determining static and structural measurements while the Villanueva-stained specimen was used for assessing fluorochrome labeling and dynamic measurements of bone formation. A saw microtome (Model 1600, Leica, Germany) was used to cut the embedded samples (2–4 *μ*m thick). Sections were analyzed semiautomatically using BIOQUANT software (R&M Biometrics, Nashville, TN, USA). Standard nomenclature was used to express the histomorphometric values. The data collection area contained only trabecular bone and bone marrow; the primary spongiosa was not included.

### 2.6. Assessment of Trabecular Bone Structures

The microarchitectural trabecular bone structure of L5 vertebra specimens was evaluated using microcomputed tomography (*μ*CT40, Scanco Medical, Switzerland). Lumbar vertebrae were scanned from the caudal to cranial end (530 slices; 16 *μ*m/slice). This region included 530 images obtained from each vertebra using the same isotropic voxel resolution and integration time as described with the tibiae. The volume of interest selected was 25 slices away from the appearance of the growth plate at each end of the vertebral body resulting in approximately 300 slices. The trabecular region was separated from the cortical bone using semiautomated contours. The trabecular bone morphometric parameters assessed with *μ*CT included the BV expressed as a percentage of total volume (BV/TV), trabecular number (Tb.N), trabecular thickness (Tb.Th), and trabecular separation (Tb.Sp). Nonmetric parameters included structure model index (SMI) which is an indicator of plate-rod arrangement of the bone structure and connectivity density (Conn.D).

### 2.7. Biomechanical Properties

L4 vertebra specimens were cleaned and freed of surrounding soft tissues. BMD was estimated using DXA, equipped with appropriate software for BMD assessment in small laboratory animals.

Biomechanical properties were assessed as described elsewhere [51]. Briefly, vertebral strength was measured at a rate of 3 mm/min using Instron 5543 (Canton, MA, USA). Vertebrae were tested in compression with force applied to the cranial and caudal surfaces. The load-displacement curve was recorded during the test. The ultimate load, yield load, and the stiffness of each specimen were measured from the load-displacement curve. Ultimate stress, yield stress, and flexural modulus of elasticity were calculated from the beam-bending equations.

### 2.8. Statistical Analysis

Statistical analysis of data involved estimation of means and standard error of the mean (SEM) for each of the groups. Analysis of variance was performed using the mixed model procedure of SAS (version 9.2, SAS Institute, Cary, NC, USA). If significant *F*-values were reported, a post-hoc test with a Tukey adjustment was performed for multiple comparison purposes. Significance was set at *P* < 0.05.

## 3. Results

### 3.1. Food Intake, Body Weight, and Uterus Weight

Even though rats were pair-fed to the Sham group, all Ovx rats had significantly higher final body weights (*P* = 0.0003). The vitamin E treatment had no effect on body weight (Table 1). Ovariectomy, as expected, caused atrophy of uterine tissue (*P* < 0.0001) confirming bilateral ovariectomy (Table 1).

### 3.2. Serum Vitamin E Levels and ALP Activity

The mean serum level of vitamin E in rats that received supplemental vitamin E was significantly higher (*P* = 0.04) than in Ovx control group (Table 1). Also, when compared to Ovx control group, MD and HD of vitamin E significantly (*P* = 0.0039) increased serum ALP activity by approximately 28 and 30%, respectively.

### 3.3. Bone Mineral Density and Content

As expected, ovariectomy significantly (*P* = 0.04) decreased whole-body BMD, but there were no differences (*P* = 0.88) in BMD among the four Ovx groups due to vitamin E treatment (Table 2). No significant (*P* = 0.28) differences were observed in whole-body BMC among treatment groups (Table 2).

### 3.4. Bone Histomorphometry

Based on bone static histomorphometry Ovx control animals lost approximately 75% of their proximal tibia cancellous bone volume (BV/TV) over the duration of the study (220 days). This loss was not ameliorated by any of the supplemental doses of vitamin E (Table 3). Ovariectomy caused an increase in the values of bone resorption parameters including osteoclast/bone surface (Oc.S/BS) and eroded surface/bone surface (ES/BS) by 76 and 79%, respectively. However, MD as well as HD of vitamin E significantly prevented these increases (Table 3).

Bone dynamic histomorphometry indicated that bone formation parameters including mineralizing surface/bone surface (MS/BS) and bone formation rate/bone volume (BFR/BV) were significantly increased due to ovariectomy (Table 3). Supplemental vitamin E at LD and MD increased MS/BS by 42 and 50%, respectively, when compared to the Ovx control group. BFR/BV was significantly increased by 65% in the group receiving the MD of vitamin E when compared to Ovx control group (Table 3).

### 3.5. Trabecular Microarchitectural Properties of L5 Vertebrae

The effects of Ovx and supplemental doses of vitamin E on trabecular microarchitectural parameters of L5 vertebra bone are presented in Table 4. Ovariectomy decreased BV/TV, Tb.N, and Tb.Th by 37, 11, and 21%, respectively. As expected, the value of Tb.Sp and Conn.D increased by 14 and 29%, respectively, due to ovariectomy. There were no effects of vitamin E treatment observed on BV/TV, Tb.N, Tb.Th, and Tb.Sp indicating that despite the favorable changes in osteoclast and osteoblast activity observed in this study, supplementation with vitamin E at this point in time had not reversed Ovx-induced alterations in trabecular bone microarchitectural parameters. The results also indicate no treatment effect of vitamin E on SMI.

### 3.6. Bone Density and Biomechanical Properties

**Figure 1:**
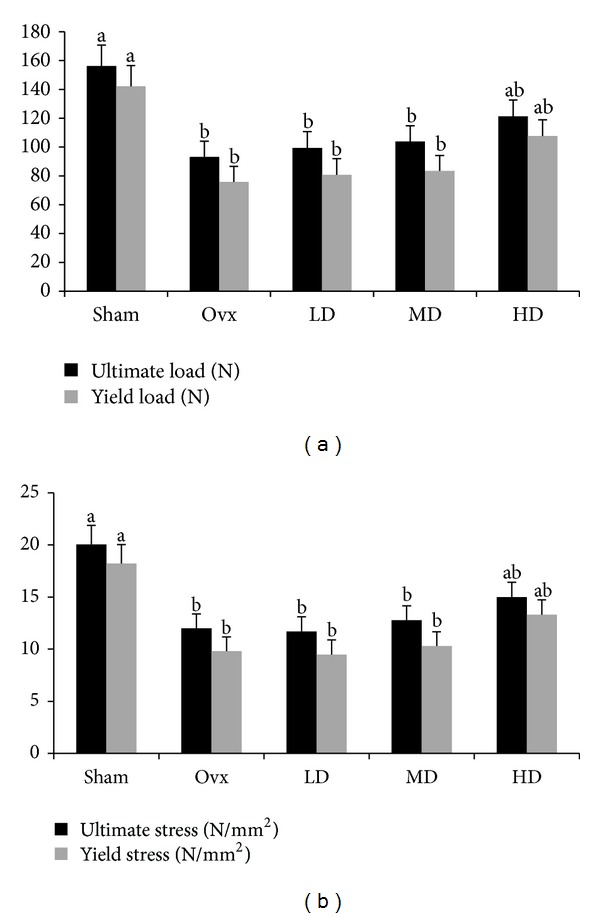
Effects of ovariectomy (Ovx) and three doses of vitamin E: low dose (LD, 300 mg/kg diet), medium dose (MD, 525 mg/kg diet), and high dose (HD, 750 mg/kg diet) on biomechanical parameters, (a) ultimate and yield load and (b) ultimate and yield stress of L4 vertebrae. Values are expressed as means ± S.E.M. Values that share the same superscript letters are not significantly (*P* ≥ .05) different; *n* = 12 rats/group.

Ovariectomy significantly (*P* < 0.0001) decreased L4 vertebra BMD; however, there were no differences in BMD among the four Ovx groups due to vitamin E treatment (Table 2). Biomechanical properties of the vertebral body such as ultimate and yield load (Figure 1(a)) and ultimate and yield stress (Figure 1(b)) were increased in the group receiving the HD of vitamin E (*P* = 0.003, *P* = 0.009, *P* = 0.003, and *P* = 0.001, resp.). Ultimately, stiffness and elastic modulus were not affected (*P* = 0.18, *P* = 0.06, resp.) by any of the treatments.

## 4. Discussion

The overall findings of our previous studies with vitamin E using rat [46, 47] and mouse [45] models of osteoporosis indicate that vitamin E is able to improve the quality of bone, especially under high-stress conditions such as unloading and aging, while unable to prevent the loss of BMD. Although BMD may at best predict the incidence of fracture by approximately 70% [52–54], other factors such as bone geometry and bone quality are of equal importance. Our data suggest that improvements in bone structural properties may occur in the absence of favorable changes in BMD. Other examples of this phenomenon reported in the literature include the observation that sodium fluoride increases BMD by nearly 10% per year while at the same time increases the rate of fracture [55, 56]. In support of our earlier observations [45, 47, 57], the findings of this study also suggest that vitamin E improves bone biomechanical properties as indicated by higher ultimate and yield load and stress while not altering BMD. Similarly, Shuid et al. [58] have shown that supplementation with vitamin E enhances femoral strength as evidenced by enhanced ultimate and yield load and stress in young male rats. However, the question remains as to why vitamin E is incapable of increasing BMD values after bone loss has occurred. A reasonable explanation for these findings may be tied to the fact that by the end of 120-day pretreatment period, too little trabecular bone remained so that an increase in bone formation induced by vitamin E, due to upregulation of osteoblasts and suppression of bone erosion by osteoclast, was unable to restore the trabecular bone structure.

When the amount of vitamin E in the diet increased from 75 mg/kg diet (control diet) to 300, we observed a significant increase in the mean serum levels of vitamin E. However, when doses of vitamin E were further increased, the effect on circulating vitamin E became indistinguishable which indicates that vitamin E level in the diet may have reached its threshold in terms of absorption or transport. This has been observed even in healthy individuals as reported by Traber and Sies [59] who stated that the efficiency of vitamin E absorption is low and the uptake decreases as the dose increases. In fact, there are studies reporting that increasing doses of vitamin E supplementation (up to 800 mg) fail to raise plasma levels of vitamin E [60, 61]. Despite this lack of an effect on circulating vitamin E, we did see differences in the response in bone. This suggests that tissue levels of vitamin E might have been altered indicating that circulating levels of vitamin E may not reflect tissue vitamin E levels [62]. Furthermore, vitamin E was able to dose-dependently increase serum ALP concentrations, a nonspecific biomarker of bone formation expressed in the osteoblastic plasma membrane. These results are supported by the findings of our previous study in which vitamin E tended to increase the levels of serum ALP [47].

In terms of bone resorption, static histomorphometry data of proximal tibiae indicate that supplementation with vitamin E reduces osteoclast activity as indicated by decreases in Oc.S/BS and ES/BS in the vitamin E-supplemented groups when compared to Ovx-control group. Thus, these findings suggest that vitamin E supplementation decreases bone resorption in the Ovx rat model. A recent report by Fujita et al. [63] suggests that vitamin E reduces bone mass in nonstressed wildtype mice or rats that had their diets supplemented with *α*-tocopherol by enhancing bone resorption and osteoclast size as seen in wildtype bone-marrow cells stimulated with receptor activator of nuclear factor kappa-B and treated with *α*-tocopherol; however, our findings are in agreement with the findings of several other studies which report the beneficial effects of vitamin E on bone. For instance, Hermizi et al. [64] have demonstrated that supplementation with vitamin E lowered Oc.S/BS and ES/BS in male rats after receiving nicotine in comparison to control group. Additionally, Ahmad et al. [65] reported that vitamin E is also able to decrease ES/BS in male rats receiving ferric nitriloacetate. Based on the aforementioned findings, one could speculate that vitamin E supplementation is only beneficial under stress conditions. However, Mehat et al. [66] have shown that supplementation with vitamin E reduces ES/BS in young male rats when compared to control group suggesting that vitamin E is also beneficial under normal conditions.

Rates of bone turnover were evaluated using dynamic histomorphometry of the proximal tibiae. MS/BS was higher in the groups supplemented with vitamin E than in the Ovx control group. In addition, supplementation with the MD of vitamin E increased bone formation (BFR/BS) by 65% when compared to Ovx control group. These results indicate that supplementation with vitamin E increases mineralization and bone formation in the Ovx rat model. These findings are supported by Mehat et al. [66] that have found higher MS/BS and BFR/BS in young male rats following supplementation with vitamin E when compared to control group.

Supplementation with vitamin E was not able to reverse the unfavorable alterations of microarchitectural parameters in the L5 vertebrae due to ovariectomy. These findings were similar to our previous findings [46] in which vitamin E did not alter BV/TV, Tb.Th, and Tb.N in Orx male rats; however, it slightly differs from one of our earlier findings [47] in which BV/TV as well as Tb.N was increased by adequate and high doses of vitamin E in hindlimb unloaded male rats when interactions between diet and unloading were evaluated. The slight difference in the findings from our laboratory may be explained by the use of histomorphometry in the latter study versus *μ*CT in the former and present study; yet, it is important to note that in this study, histomorphometry as well as *μ*CT data are in agreement. Contrary to our findings, Shuid et al. [58] have shown that supplementation with vitamin E significantly increases BV/TV, Tb.Th, and Tb.N and decreases Tb.Sp. Since supplemental vitamin E bioavailability is greatly dependent on the form it is presented to the intestine absorptive cells, this difference may be, in part, attributed to the distinct methods of supplementation utilized in each study. While Shuid et al. [58] administered 60 mg/kg body weight/day by oral gavage, we fed the animals a diet containing 300 (LD), 525 (MD), and 750 (HD) mg of vitamin E/kg diet. In addition, Shuid et al. [58] were investigating the ability of vitamin E prevent bone loss using normal male rats whereas our study was examining the role of vitamin E in reversing bone loss due to ovarian hormone deficiency using Ovx rats.

One of the mechanisms by which vitamin E affects bone health is through its anti-inflammatory and antioxidative properties. The role of vitamin E in these processes has been clearly identified and is significant in the prevention of chronic diseases including atherosclerosis [67, 68] and cancer [69]. Macrophages are involved not only in the initiation but also in the regulation of inflammatory and immune responses [18, 70–72]. The increased secretion of proinflammatory molecules from macrophages such as PGE_2_, IL-6, TNF-*α* [73], and IL-1 [9] may lead to an increase in the rate of bone resorption, hence, accelerating bone loss. For instance, vitamin E did not suppress the synthesis of PGE_2_ and IL-6 in lipopolysaccharide-stimulated macrophages [74]. These findings suggest that vitamin E may act differently under various stress conditions. In terms of bone, this may partially explain the contrasting findings in Ovx and Orx rats compared to hindlimb unloaded rats. Although a few studies have reported that vitamin E positively influences bone quality [47, 58, 64], these studies examined the role of vitamin E in preventing bone loss but not its reversal. In conclusion, our observations as well as those of other investigators suggest that vitamin E supplementation is able to prevent bone loss and improve bone biomechanical properties even though it is unable to reverse the loss of bone due to ovarian hormone deficiency.

## Figures and Tables

**Table 1:** Effects of ovariectomy (Ovx) and three doses of vitamin E: low dose (LD, 300 mg/kg diet), medium dose (MD, 525 mg/kg diet), and high dose (HD, 750 mg/kg diet) on body and tissue weights, serum vitamin E, and serum ALP activity.

Parameters	Sham	Ovx	LD	MD	HD
Body Weight (g)					
Initial	299.3 ± 11.8	307.9 ± 8.9	310.4 ± 9.2	307.7 ± 8.9	308.6 ± 8.9
Final	328.0 ± 20.19^a^	409.8 ± 11.27^b^	406.0 ± 10.37^b^	392.9 ± 10.35^b^	394.9 ± 8.51^b^
Uterine weight (g)	0.710 ± 0.035^a^	0.141 ± 0.027^b^	0.209 ± 0.031^b^	0.144 ± 0.029^b^	0.158 ± 0.028^b^
Vitamin E (mg/mL)	26.78 ± 2.96^ab^	22.47 ± 2.24^b^	31.32 ± 2.41^a^	29.51 ± 2.24^a^	30.98 ± 2.32^a^
ALP (U/L)	38.63 ± 2.59^a^	46.79 ± 2.89^a^	56.54 ± 3.68^a^	60.29 ± 5.64^b^	61.23 ± 4.46^b^

Values within a row that share the same superscript letters are not significantly (*P* ≥ .05) different. Values are means ± S.E.M.

ALP: alkaline phosphatase.

**Table 2:** Effects of ovariectomy (Ovx) and three doses of vitamin E: low dose (LD, 300 mg/kg diet), medium dose (MD, 525 mg/kg diet), and high dose (HD, 750 mg/kg diet) on DXA bone parameters of whole body and fourth lumbar vertebrae (L4).

Parameters	Sham	Ovx	LD	MD	HD
Whole body (g)					
Baseline					
BMC (g)	10.3 ± 0.38	10.55 ± 0.20	10.84 ± 0.18	10.57 ± 0.22	10.73 ± 0.21
BMD (g/cm^2^)	0.1774 ± 0.0029	0.1811 ± 0.0030	0.1806 ± 0.0013	0.1790 ± 0.0020	0.1799 ± 0.0018
Final					
BMC (g)	11.14 ± 0.40	11.72 ± 0.22	11.98 ± 0.16	11.59 ± 0.26	11.8 ± 0.20
BMD (g/cm^2^)	0.1734 ± 0.0029^a^	0.1657 ± 0.0021^a^	0.1635 ± 0.0013^ab^	0.1649 ± 0.0020^a^	0.1644 ± 0.0018^ab^
L4 vertebra					
BMD (g/cm^2^)	0.240 ± 0.005^a^	0.199 ± 0.004^b^	0.202 ± 0.004^b^	0.199 ± 0.004^b^	0.199 ± 0.004^b^

Values within a row that share the same superscript letters are not significantly (*P* ≥ .05) different. Values are means ± S.E.M.

BMC: bone mineral content*; *BMD: bone mineral density.

**Table 3:** Effects of ovariectomy (Ovx) and three doses of vitamin E: low dose (LD, 300 mg/kg diet), medium dose (MD, 525 mg/kg diet), and high dose (HD, 750 mg/kg diet) on the proximal tibial metaphysis cancellous bone cellular activity.

Parameters	Sham	Ovx	LD	MD	HD
BV/TV	12.010 ± 1.526^a^	3.020 ± 1.649^b^	2.470 ± 1.238^b^	1.865 ± 1.238^b^	2.022 ± 1.238^b^
Bone resorption parameters					
Oc.S/BS (%)	1.276 ± 0.325^a^	2.252 ± 0.347^b^	1.841 ± 0.347^ab^	1.265 ± 0.347^a^	1.008 ± 0.347^a^
ES/BS (%)	1.473 ± 0.375^a^	2.651 ± 0.393^b^	1.885 ± 0.393^a^	1.612 ± 0.393^a^	1.218 ± 0.393^a^
Bone formation parameters					
MS/BS (%)	4.842 ± 2.144^a^	13.97 ± 2.23^b^	19.942 ± 2.236^c^	21.002 ± 2.236^c^	13.137 ± 2.236^b^
BFR/BV (%/yr)	0.117 ± 0.370^a^	1.295 ± 0.380^b^	1.909 ± 0.380^bc^	2.148 ± 0.380^c^	1.456 ± 0.380^bc^

Values within a row that share the same superscript letters are not significantly (*P* ≥ .05) different. Values are means ± S.E.M.

BV/TV: bone volume as percentage of tissue volume; Oc.S/BS: osteoclast/bone surface; ES/BS: eroded surface/bone surface; MS/BS: mineralizing surface/bone surface; BFR/BV: bone formation rate/bone volume.

**Table 4:** Effects of ovariectomy (Ovx) and three doses of vitamin E: low dose (LD, 300 mg/kg diet), medium dose (MD, 525 mg/kg diet), and high dose (HD, 750 mg/kg diet) on three-dimensional (3D) microcomputed tomography (*μ*CT) structural parameters of fifth lumbar vertebrae.

Parameters	Sham	Ovx	LD	MD	HD
BV/TV (%)	0.382 ± 0.023^a^	0.242 ± 0.023^b^	0.208 ± 0.023^b^	0.217 ± 0.023^b^	0.230 ± 0.023^b^
Tb.N (1/mm)	3.290 ± 0.150^a^	2.920 ± 0.150^ab^	2.660 ± 0.150^b^	2.560 ± 0.150^b^	2.900 ± 0.150^ab^
Tb.Th (mcm)	0.109 ± 0.003^a^	0.087 ± 0.003^b^	0.084 ± 0.003^b^	0.088 ± 0.003^b^	0.084 ± 0.003^b^
Tb.Sp (mcm)	0.295 ± 0.018^a^	0.339 ± 0.018^ab^	0.378 ± 0.018^b^	0.387 ± 0.018^b^	0.345 ± 0.018^ab^
Conn.D (1/mm^3^)	25.8 ± 2.5^a^	33.4 ± 2.5^b^	29.5 ± 2.5^ab^	25.3 ± 2.5^a^	34.1 ± 2.5^b^
SMI	1.158 ± 0.258^a^	0.295 ± 0.258^b^	0.640 ± 0.258^b^	0.472 ± 0.258^b^	0.427 ± 0.258^b^

Values within a row that share the same superscript letters are not significantly (*P* ≥ .05) different. Values are means ± S.E.M.

BV/TV: bone volume as percentage of tissue volume; Tb.Th: trabecular thickness; Tb.N: trabecular number; Tb.Sp: trabecular separation; Conn.D: connective density; SMI: structure model index.
